# Innovative engineering of scalable, renewable and spherical organic nanoparticles for high fire safety, UV protection and antibacterial properties of polyvinyl alcohol nanocomposites films

**DOI:** 10.1038/s41598-024-80360-y

**Published:** 2024-11-21

**Authors:** Nour Fathi Attia, Mohamed A. Nour, Sally E. A. Elashery

**Affiliations:** 1 Gas Analysis and Fire Safety Laboratory, Chemistry Division, National Institute for Standards, 136, 12211 Giza, Egypt; 2 Chemistry Department, Faculty of Science, Cairo University, Gamaa Str, Giza, 12613 Egypt

**Keywords:** Sustainability, Green flame retardants, Organic nanoparticles, Safe antibacterial, Polymer nanocomposites, UV protection, Environmental chemistry, Materials chemistry, Supramolecular chemistry, Surface chemistry, Biotechnology, Chemistry, Materials science, Nanoscience and technology

## Abstract

A novel and environmentally friendly route was developed for production of sustainable flame retardant, antibacterial and UV protective nanoparticles for polymeric films nanocomposites. For the first time, dried molokhia leaves were engineered into spherical nanoparticles with an average size of 8.5 nm via an eco-friendly, one-pot solid-state ball-milling method. The engineered nanoparticles were proved using spectroscopic and microscopic techniques. The sustainable nanoparticles were employed as an efficient and green flame retardant, antibacterial and UV protective materials for polyvinyl alcohol (PVA) nanocomposite films. The distinct compatibility between PVA chains and spherical nanoparticles afford excellent homogeneous dispersion of each nanoparticle in the polymer matrix. Compared to blank PVA film which burned at a rate of 125 mm/min, the novel nanoparticles achieved significant flame retardancy for polymer nanocomposites films recording zero rate of burning. Their outstanding charring ability and naturally doped elemental composition were attributed to their higher flame retardancy achieved. Moreover, the newly developed multifunctional nanoparticles integrated outstanding UV protection feature to developed polymer nanocomposite films recording UV protection factor superiority of more than 900% compared to nanoparticle free film. Noteworthy to note that, the nanoparticles afford excellent inhibition to bacterial growth against *Escherichia coli and Staphylococcus aureus* over the surface of developed polymer nanocomposite films achieving clear inhibition zone of 9 and 7.6 mm compared to zero mm for pristine polymer film, respectively. In addition, a proposed and clarified flame retardancy mechanism was presented. Additionally, an assessment was conducted regarding the economic feasibility of producing sustainable multifunctional nanoparticles on an industrial scale.

**Supplementary Information:**

The online version contains supplementary material available at 10.1038/s41598-024-80360-y.

## Introduction

Nowadays, organic polymers are emerging as viable alternatives to conventional inorganic and are becoming more and more important in a variety of disciplines. However, these natural polymers are flammable by nature, hence, it is necessary to reduce their flammability hazards especially in industrial applications where materials must have flame retardant properties^1–4^. On the other hand, polyvinyl alcohol (PVA) is very interesting organic polymer with outstanding features such as high transparency, excellent film forming properties^5–9^, thus, it is used in food packaging and separation applications^10–13^. This is in addition to other high-tech application^14,15^. Nevertheless, the high flammability hazards of PVA restricts its usage in various industrial applications^16–19^. Therefore, it is mandatory to incorporate flame retardants materials as dispersed fillers in PVA matrix to reduce the flammability properties and enhance its fire safety^20,21^. Various flame-retardant materials have been implemented for enhancing fire safety for PVA utilizing traditional organic and inorganic phosphate-based materials^22–25^. However, recently nanobased and bioderived materials have been introduced as flame retardant materials for PVA, however, the energy intensive synthesis process and cost of raw materials is considering an obstacle issue for their scalable production^21,26–28^. Interestingly, peculiar structure of PVA (molecular chain rich with hydroxyl groups) afford easier incorporation of flame-retardant fillers inside polymer matrix of PVA. Nevertheless, the wide use of PVA in food packaging systems and medical applications was limited due to the easier bacterial growth on its surface in conjunction with recent pandemic (virus) outbreak. Additionally, due to its organic nature its thin film is significantly exposed to partial degradation by exposure to high energy UV rays. Hence, to address these obstacles and increase the range of industrial applications for PVA, antibacterial and UV protective materials should be incorporated in conjunction to flame retardant materials^29^. On the other hand, molokhia is grown in large quantities every year and is regarded as a popular vegetable in Egypt. Furthermore, because of the abundance of various phenolic and antioxidant chemicals in its extract (ME), it has been discovered to be a very promising material in a range of applications^30–33^. Recently, we have proved the ME is very promising as flame retardant and UV protective material for textile fabrics due to their special composition^34^. Thus, design and engineering of scalable, green, sustainable and cost-effective multifunctional flame retardant, antibacterial and UV protective nanoparticles for PVA matrix is crucial and industrial requirement. In this study, scalable flame retardant, antibacterial and UV protective spherical nanoparticles were developed from green precursor. Molokhia leaves nanoparticles (MLNPs) were synthesised via one-pot environmentally friendly method and then exploited as green flame retardant, antibacterial and UV protective for PVA nanocomposites. Also, the dispersion of developed nanoparticles in PVA nanocomposites were evaluated. Also, the flammability properties, mechanical properties, thermal stability, antibacterial and UV protection of developed nanocomposite films were thoroughly studied.

## Experimental section

### Materials

Polyvinylalchol (PVA) was bought from El Naser Pharmaceutical Chemicals, Cairo, Egypt. Green Molokhia leaves (ML) were collected from local Egyptian market. The deionized water (DI) was used for synthesis process.

### Synthesis of molokhia leaves nanoparticles (MLNPs)

First of all, molokhia leaves were removed from the molokhia stems and washed and then dried in sun for 7 days. This followed by grinding of ML to fine powder. Then, 10 g of molokhia leaves powder (ML) was put in a ball-milling ceramic capsule and the ball-milling process was conducted for 24 h at 300 rpm. The obtained molokhia leaves nanoparticles was collected and sieved and be ready for next step. The attained molokhia leaves nanoparticles was denoted as MLNPs.

### Synthesis of polyvinylalchol-MLNP nanocomposites (PVA-MLNPs)

In glass beaker, 5 g of PVA was completely dissolved in 100 ml DI water with magnetic stirring. Then, various masses of developed MLNPs (10,20,40 and 50 wt%) was dispersed individually with magnetic stirring for 1 h and followed by ultrasonication for 10 min as indicated in Table 1. The developed nanocomposites solution was poured in aluminium foil template and water was evaporated and the obtained nanocomposite films were obtained and dried at 70 °C. The attained nanocomposites were denoted as PVA-MLNPX as PVA refers to polymer and MLNPs refers to molokhia leaves nanoparticles and X refers to mass of MLNP in wt% dispersed in PVA matrix (Table 1).

**Table 1:** Composition of engineered polymer nanocomposites.

Sample Code	PVA (wt%)	MLNP (wt%)
PVA-MLNP-0 PVA-MLNP-10	100 90	0 10
PVA-MLNP-20	80	20
PVA-MLNP-40	60	40
PVA-MLNP-50	50	50

### Characterization

The chemical structure of ML, MLNPs and their corresponding nanocomposite films were carried out using FT-IR spectroscopy analysis in the spectral range of 4000 –400 cm^− 1^ by Thermo Scientific’s Nicolet 380 spectrophotometer. Scanning electron microscope (SEM) photographs of the surface morphology of char layer of burned PVA and it nanocomposite were acquired using a scanning electron microscope (Quanta FEG-250, operating at a voltage of 20 kV). Moreover, the dispersion of developed nanocomposites was studied using Transmission electron microscope images obtained using TEM; JEOL-JSM-1400plus. Utilising a Shimadzu UV 3101PC UV-Vis Spectrophotometer, the UPF values for PVA and its derived nanocomposite films were investigated. The thermogravimetric analysis was carried out using DTG 60, under nitrogen atmospheric flow, from room temperature to 750 °C with a heating rate of 10 °C/min. The fire hazards of blank PVA and developed nanocomposites were evaluated using rate of burning via UL94 (horizontal test) according to IEC 60695-11-10 standard^35^. Moreover, the inhibition of bacterial growth against *Staphylococcus aureus* and *Escherichia coli* bacteria was measured consistent with AATCC standard test method 147–2004^36^. The average clear inhibition zone (W), where W = (T-D)/2, from three replicates was used to examine the samples. T is the overall diameter of the test specimen and clear zone in mm, W is the width of the clear inhibition zone in mm, and D is the width of the test specimen itself in mm.

## Results and discussion

### Innovative synthesis process of MLNPs and their characterization

**Fig. 1:**
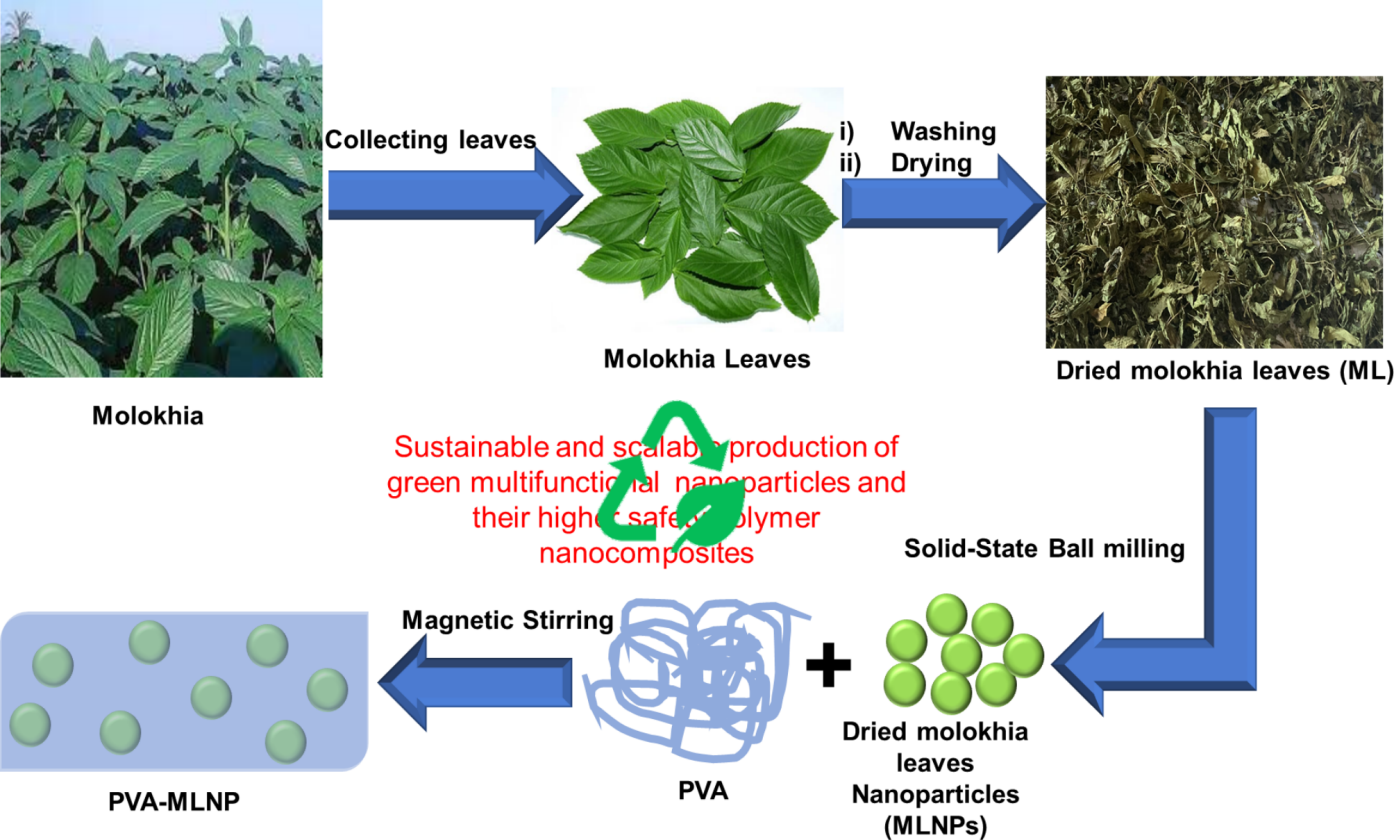
Schematic diagram representing the green synthesis process of sustainable spherical MLNPs and their well dispersed PVA nanocomposites.

Green and innovative approach was employed for the first time for production of scalable spherical nanoparticles from green molokhia leaves (ML) via one pot solid-state ball-milling process as described in Fig. 1. The green ML was washed and dried and then ball-milled for 24 h in solvent-less process under 300 rpm at ambient conditions. The obtained nanoparticles were denoted as MLNPs. The engineered nanoparticles were produced in scalable quantity with spherical shape and narrow size distribution as indicated below. Furthermore, the MLNPs were utilized as green and biosafe flame retardant, antibacterial and UV protective nanofillers for PVA nanocomposites, thus expanded their potential industrial applications such as flexible solar cells, food packaging and wearable devices.

**Fig. 2:**
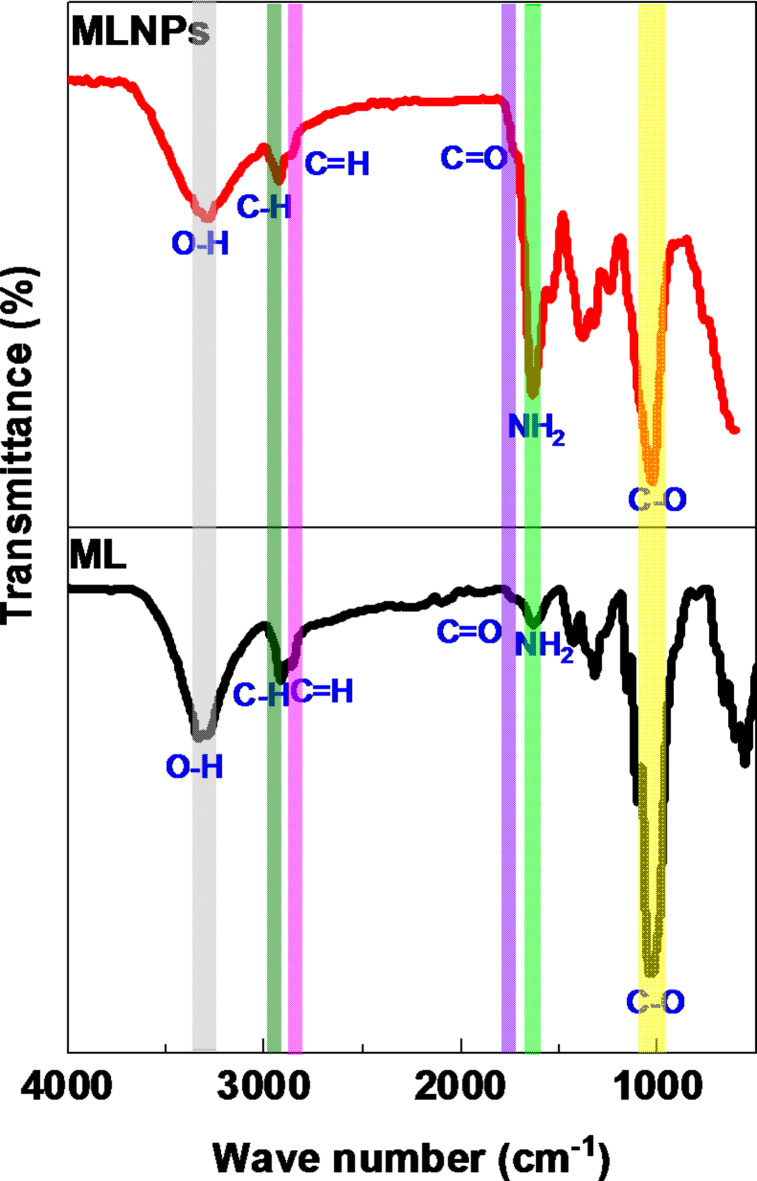
FT-IR spectra of dried ML and MLNPs.

The chemical structure of developed MLNPs were elucidated using FT-IR spectroscopy and compared to the un ball-milled traditional ML to validate the synthesis approach. The characteristic absorption peaks of dried ML, which are the same as ME, are shown in Fig. 2^37^. The absorption peak noticed at 3300 cm^− 1^ is assigned to stretching vibration of O-H, however, the one detected at 1730 is ascribed to stretching vibration of C = O, these findings confirm the presence of antioxidants in ML^37–40^. The absorption peaks positioned at1036 and 1640 cm^− 1^ are attributed to stretching vibration of C-O and free amine (-NH_2_) group of dried ML respectively^34,37–40^. Additionally, the absorption peaks situated at 2915 and 2849 cm^− 1^ are recognised to stretching vibration of C-H and C = H groups respectively^34,38–40^. This validates the existence of various antioxidants such as 5-caffeoylquinic acid, 3,5-dicaffeoylquinic acid, ascorbic acid, α-tocopherol, β -carotene and glutathione in dried ML^34,37–40^. For MLNP which developed at 24 h, similar characteristic absorption peaks were found, thus, the absorption peaks detected at 2920 and 2852 cm^− 1^ are attributed to stretching vibration of C-H and C = H groups respectively^34,37–39^, however, the absorption peak noticed at 1733 cm^− 1^ is ascribed to stretching vibration of C = O^37^. Moreover, the absorption peaks positioned at 3286, 1640 and 1031 cm^− 1^ are characteristic to stretching vibration of O-H, free amine (-NH_2_) and C-O groups, respectively^34,37–40^. This corroborated the existence of all functional groups existed in ML (5-caffeoylquinic acid, 3,5-dicaffeoylquinic acid, ascorbic acid, α-tocopherol, β -carotene and glutathione) without any change in MLNPs as indicated in Fig. 2. Thus, affirms the success of solid-state ball-milling process for production of spherical nanoparticles derived from ML (MLNPs).

**Fig. 3:**
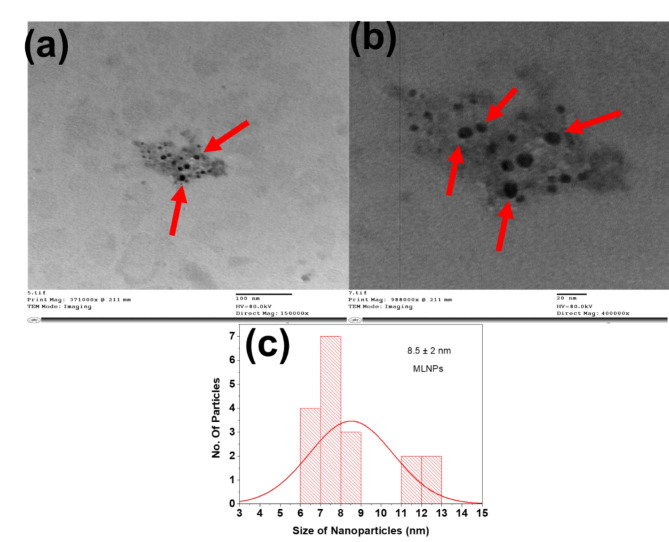
TEM image of (**a**) MLNPs, (**b**) TEM image of MLNPs at high magnification and (**c**) histogram of MLNPs.

On the other hand, the morphological properties (size & shape and distribution ) of the obtained MLNPs were evaluated using microscopic tools. Figure 3a represents the TEM image of MLNPs which displayed spherical shape of nanoparticles visualized in well dispersed form and the average size of MLNPs was found to be 8.5 nm. These morphological features of developed MLNPs were clarified and highlighted at high magnification TEM image which reflects uniform dispersion and affirms the formation of spherical nanoparticles from ML(Fig. 3b). Also, their narrow size distribution was elucidated as shown histogram graph (Fig. 3c).

### Green synthesis process of PVA-MLNP nanocomposite films and their morphological and thermal properties

**Fig. 4:**
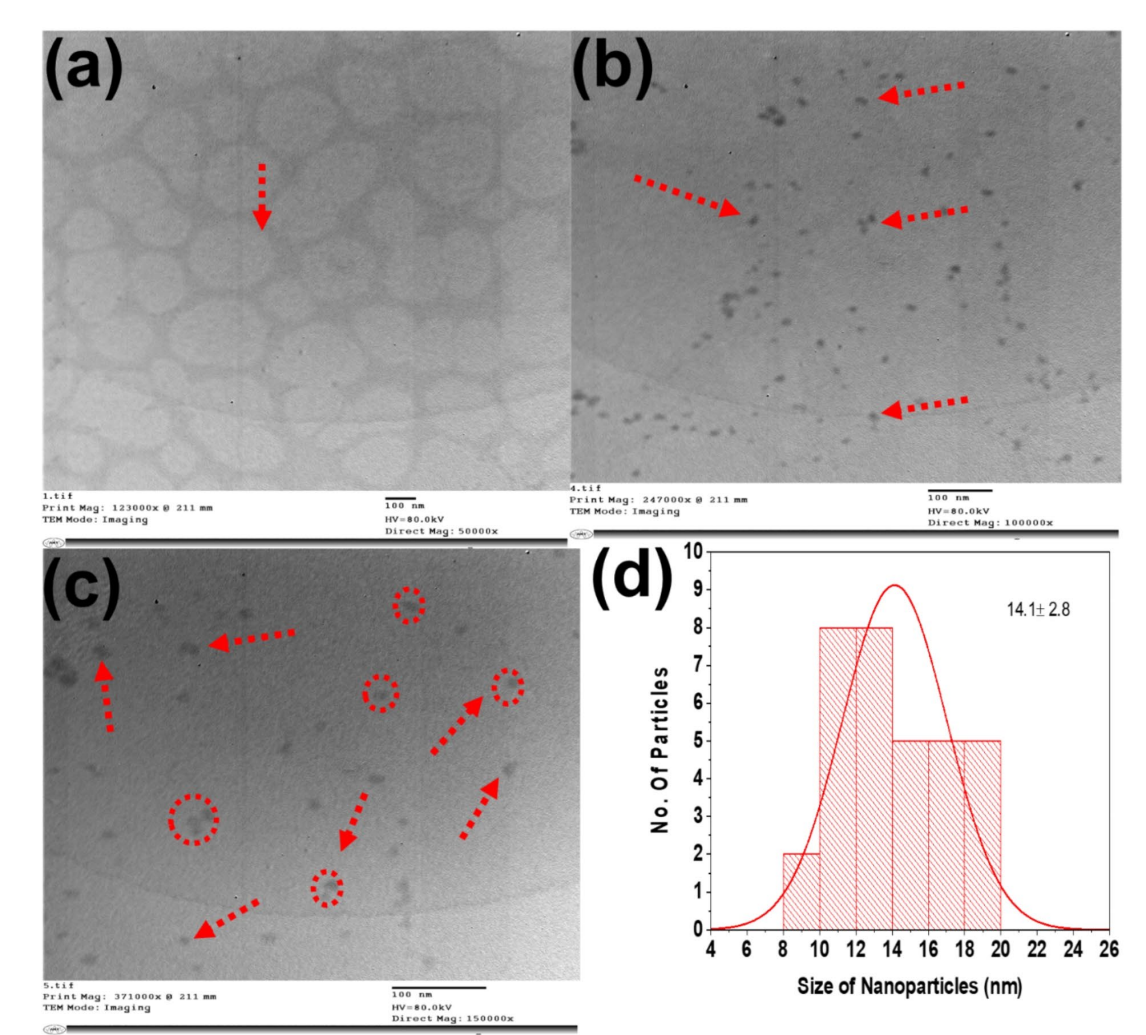
TEM image of (**a**) PVA, (**b**) PVA-MLNP-40, (**c**) PVA-MLNP-40at high magnification and (**d**) histogram of MLNPs in PVA-MLNP-40.

Once spherical MLNPs were prepared and their structure and nanoscale morphology were elucidated, they uniformly dispersed in PVA matric yielding PVA nanocomposites in the form of flexible free-standing film nanocomposites (Fig. S1). This uniform dispersion of newly developed biosafe MLNPs in PVA matrix was executed using facile magnetic stirring process of MLNPs in PVA solution for 1 h at ambient condition of temperature and pressure and followed by ultrasonication (Fig. 1). Moreover, the mass loadings of MLNPs were alternated from 10, 20, 40 and 50 wt% as depicted in Table 1. Noteworthy to note that, the driving force of magnetic stirring process provides good mechanochemical energy which affords good dispersion of spherical MLNPs inside of PVA matrix yielding PVA-MLNPs. Additionally, the good compatibility between the chemical nature of MLNPs and PVA affords good dispersion of MLNPs inside PVA chains. Additionally, due to the excellent compatibility between PVA chains and MLNPs structure and aid of magnetic stirring and ultrasonication process layers of PVA were wrapped on MLNPs surface displaying strong interfacial adhesion between them and forming of well dispersed nanocomposites. To elucidates this phenomenon microscopic morphological characterization of nanocomposites was investigated using TEM. Figure 4a represents TEM image of blank PVA chains which reflects entangled polymers chains. However, after incorporation of MLNPs in PVA matrix, uniform dispersion of spherical MLNPs were visualized in PVA-MLNP-40 nanocomposite as highlighted with red arrows (Fig. 4b). Moreover, the uniform dispersion of individual nanoparticles of MLNPs inside PVA and wrapping of their chains on the surface of MLNPs were further corroborated at high magnification TEM image and histogram of the dispersed MLNPs. Figure 4c visualized the uniform dispersion of MLNPs wrapped with PVA chains yielding bigger size of nanoparticle of an average size of 14.1 nm (Fig. 4d) compared to 8.5 nm size for MLNPs alone (Fig. 3). This afforms the successful synthesis process of PVA-MLNPs nanocomposites with excellent interfacial adhesion between MLNPs and PVA chains (Fig. 4).

**Fig. 5:**
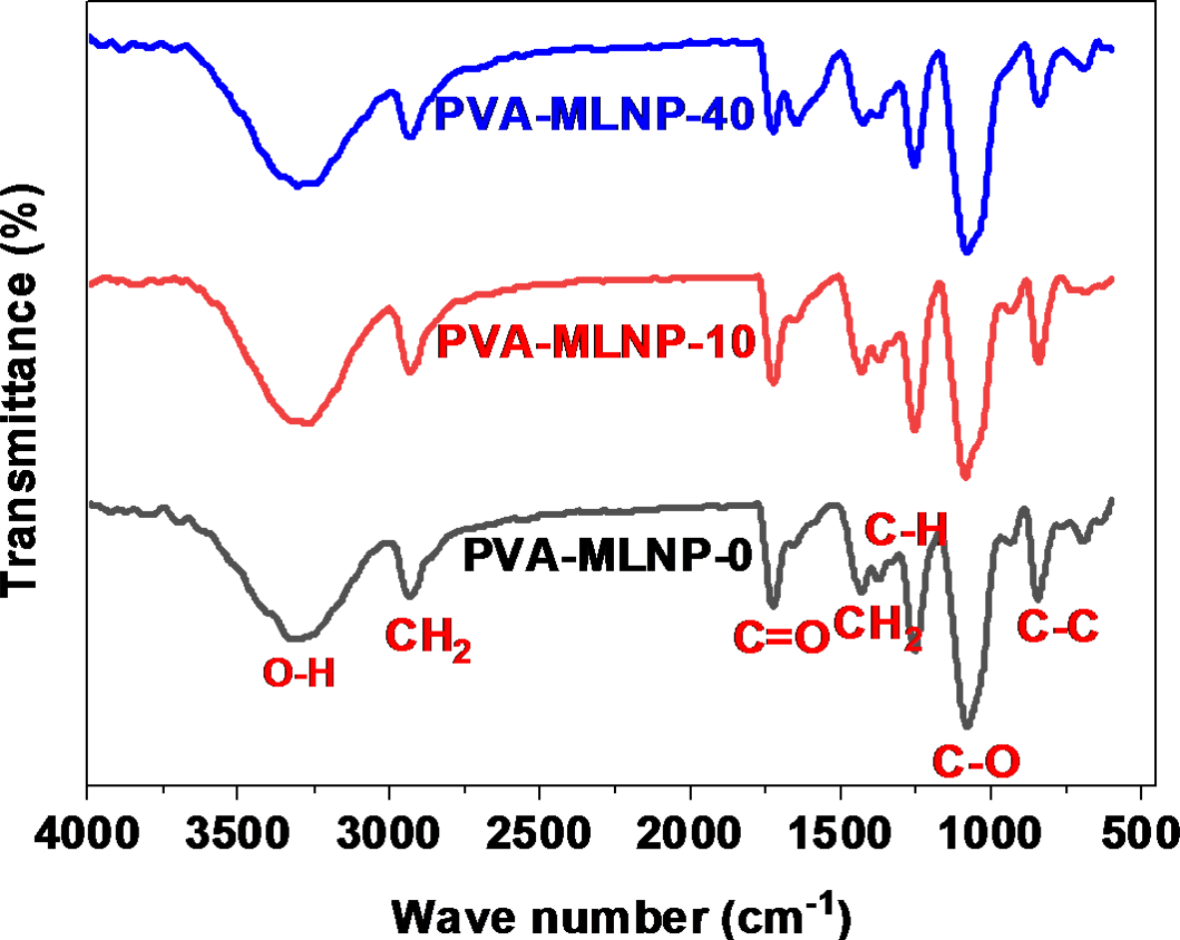
FT-IR spectra of PVA-MLNP-0, PVA-MLNP-10 and PVA-MLNP-40 films.

Interestingly, the compatibility and interaction between PVA chains and MLNPs were evaluated using FT-IR of the PVA-MLNP-0 and their corresponding nanocomposite films PVA-MLNP-10 and PVA-MLNP-40 as described in Fig. 5. Thus, for PVA-MLNP-0 the absorption peaks detected at 1720, 2932, 1430 and 1327 cm^− 1^ are assigned to stretching vibration of C = O, asymmetric stretching vibration of CH_2_, bending vibration of CH_2_ and deformation of C-H, respectively^41–43^. Additionally, the peaks positioned at 1078 and 3290 cm^− 1^ are ascribed to stretching vibration of C-O and O-H, respectively^41–43^. Moreover, the peak noticed at 841 cm^− 1^coressponds to stretching vibration of C-C^41–43^. This reflects the characteristic absorption peaks for PVA. The FT-IR spectra of PVA-MLNP-10 and PVA-MLNP-40 displayed the characteristic absorption peaks of both PVA and MLNPs, the peaks of MLNPs were overlapped by the strong absorption peaks of PVA. However, it is noticed that the intensity of characteristic absorption peaks of MLNPs increased in PVA-MLNP-40 (Fig. 5). Interestingly, inspection the FT-IR of newly developed nanocomposites, revealed that no new bonds were formed (Fig. 5). However, higher absorption shift of the stretching vibration peaks of O-H from 3290 in PVA-MLNP-0 to 3300 cm^− 1^ in PVA-MLNP-40, C = O from 1720 cm^− 1^ in PVA-MLNP-0 to 1725 cm^− 1^ in PVA-MLNP-40 and C-O from 1078 cm^− 1^ in PVA-MLNP-0 to 1086 cm^− 1^ in PVA-MLNP-40 were observed. This change was ascribed to the formation of hydrogen bonding between MLNPs surface functions and PVA chains, corroborating the formation of supramolecular interactions between MLNPs surface and PVA chains.

**Fig. 6:**
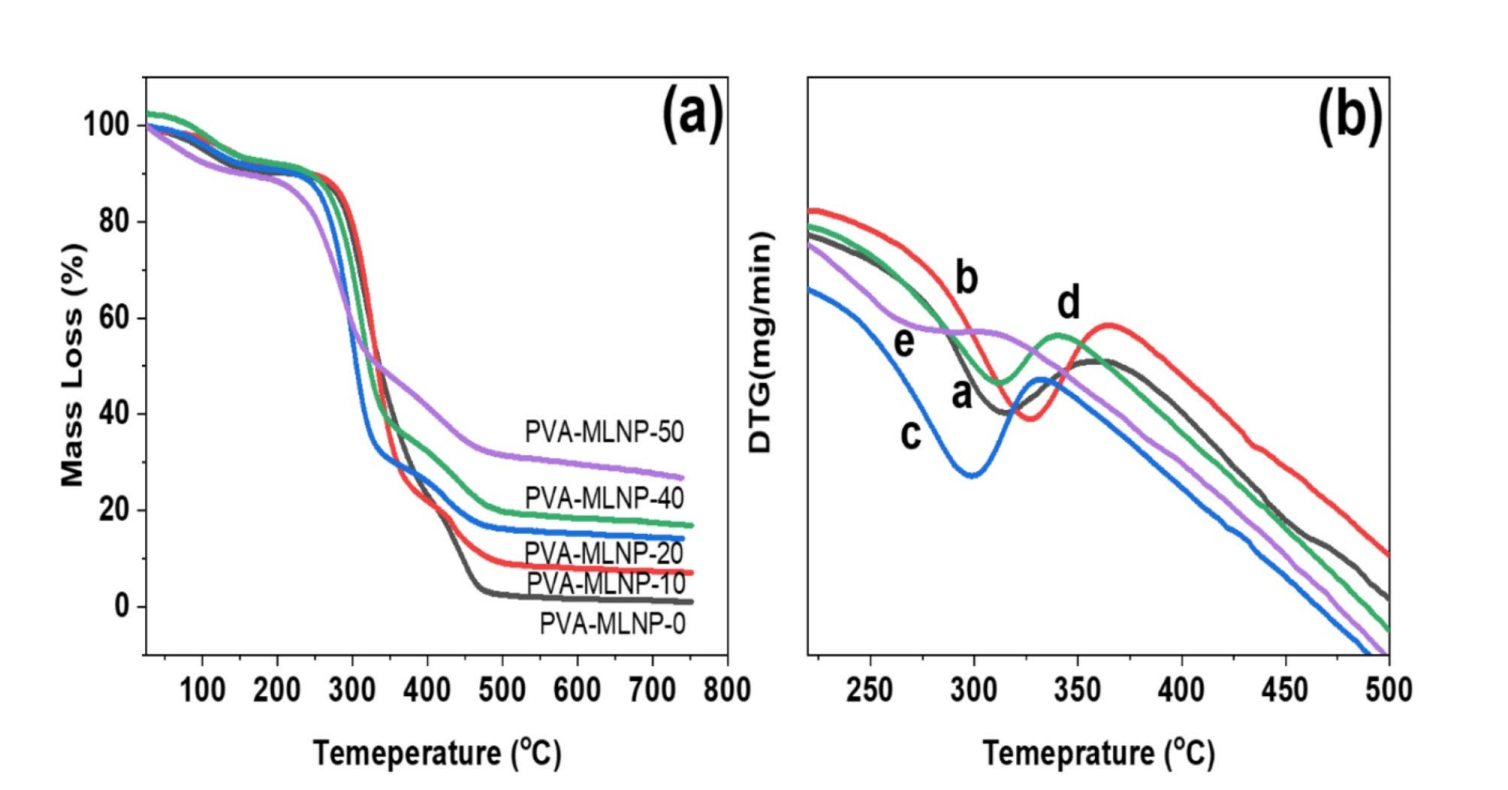
(**a**) TGA graphs of PVA-MLNP-0 and their derived nanocomposites and (**b**) DTG curves of (**a**) PVA-MLNP-0, (**b**) PVA-MLNP-10, (**c**) PVA-MLNP-20, (**d**) PVA-MLNP-40 and (**e**) PVA-MLNP-50.

On the other hands, the thermal stability of the PVA and their developed nanocomposites were evaluated using thermogravimetric analysis and data were tabulated in Table 2 and thermographs were presented in Fig. 6. Figure 6a represents the thermograph of PVA which displayed two main decomposition steps, the first one initiated at 260 °C (T_onset1_) (onset decomposition temperature) and reflected maximum mass loss at temperature (T_max_) of 315 °C (Fig. 6b) which corresponding to the release of hydroxyl groups throughout the PVA chains. However, the second decomposition step was started at (T_onset2_) 420 °C (Fig. 6b) and assigned to the decomposition of PVA polymer chains leaving char residue of 1.05 wt% at 750 °C (Table 2). Interestingly, upon incorporation of MLNPs in PVA-MLNP-10 nanocomposite similar decomposition steps were attained however, the T_onset1_ and T_max_ were increased recording 265 and 326 °C (Fig. 6b), also (T_onset2_) was shifted to higher temperature recording 426 °C leaving higher char residue of 7 wt% with lower mass loss rate as indicated in Fig. 6b. This noticed good thermal stability and flame retardancy effect of developed nanocomposite was attributed to the intrinsic ability of MLNPs for inducing PVA chains for forming protective char layer on the surface of PVA nanocomposite film instead of thermal pyrolysis of PVA chains. Thus, the protective char layer significantly reduces the mass loss rate (Fig. 6b) and inhibits the full thermal decomposition of PVA chains and in turns superior char yield was attained of 7 wt% compared to 1.05 for PVA alone (free MLNPs) (Fig. 6a). Interestingly to note that, the charring ability of MLNPs was found to be superior in the nanoscale size and their well dispersion in PVA also significantly improved their efficiency^34^. This good thermal stability and charring agent phenomena of MLNPs were further improved once the mass loadings of MLNPs was increased to 20 wt% (PVA-MLNP-20 nanocomposite) recording two-fold increase in char yield of 14.2 wt% (Table 2). Also, further increase of MLNPs mass loadings in nanocomposite records higher charring efficiency of developed nanocomposites and achieving char residue of 26.8 wt% in PVA-MLNP-50 nanocomposite achieving superior thermals stability with significant reduction in mass loss rate as indicated in Fig. 6. This corroborates the excellent flame retardancy and thermal stability properties of developed MLNPs as new multifunctional, scalable bio-safe flame retardant nanoparticles for PVA and other polymeric materials.

**Table 2:** Thermogravimetric data of pristine and polymer nanocomposites.

Sample code	T_Onset1_ °C	T_max_ °C	T_Onset2_ °C	Char residue (%)
PVA-MLNP-0	260	315	420	1.05
PVA-MLNP-10	265	326	426	7.0
PVA-MLNP-20	243	298	419	14.2
PVA-MLNP-40	253	311	420	16.7
PVA-MLNP-50	214	277	425	26.8

T_onset1_: the onset decomposition temperature of first main mass loss; T_max_: the temperature at maximum mass loss of main mass loss; T_onset2_ : the onset decomposition temperature of secondary mass loss; Char residue; is the leaving char residue at 750 °C.

### Flammability properties of developed nanocomposites

The flammability properties of the PVA (PVA-MLNP-0) and their developed nanocomposites were studied based on measuring the rate of burning of developed free standing nanocomposites films using UL94 *(Horizontal test)* according to IEC 60695-11-10 standard^35^. The rate of burning of blank free-standing film of PVA (PVA-MLNP-0) was found to be 125 mm/min as it burned quickly once exposed to flame source recording only 36 s time to afford burning of all sample distance of 75 mm as burned distance. On contrast, after dispersion of spherical MLNPs in PVA matrix of PVA-MLNP-10 nanocomposite film a significant reduction in rate of burning was found recording 54% reduction (Table 3) and the sample takes more than two-fold times compared to PVA (79 s) to burn similar burned distance (Table 3). This higher flame retardancy effect was ascribed to the good charring effect of MLNPs due to their unique chemical composition (naturally rich with nitrogen compounds)^33,34^ which trigger PVA chains for protective char layer formation on the surface of PVA-MLNPs rather than thermal decomposition^40,44–46^. This is in addition to the natural existence of precious elemental composition which played role in the flame retardancy action (Table 4). Additionally, the uniform dispersion of MLNPs and their excellent compatibility and interfacial adhesion with PVA matrix enhanced their charring effect for stimulates PVA for forming char barrier. Thus, the protective char layer slowdown the pyrolysis rate of PVA chains and isolates flaming zone from pyrolysis one which in turn retards the mass and heat transfer^33,34,44–46^. This flame retardancy effect was enhanced once mass loading of MLNPs was increased (PVA-MLNP-20) recording significant reduction in rate of burning (31 mm/min) recording reduction by 75%. This is clearly noticed in higher burning time of 117 s for 60 mm as burned distance and then affords self-extinguish of sample. This phenomenon was enhanced once 40 wt% of MLNPs was uniformly dispersed in PVA (PVA-MLNP-40) achieving self-extinguishing of sample after burning of only 25 mm of the sample. Interestingly, upon incorporation of 50 wt% of MLNPs in PVA-MLNP-50 nanocomposite higher fire safety was attained recording zero rate of burning of nanocomposite and the sample was self-extinguished before standard mark (Fig. S2). This affirms the excellent flame retardancy of MLNPs and their superior charring effect which stemmed from their uniform dispersion in polymer matrix which led to higher area of interaction (good interfacial adhesion with PVA chains) with PVA chains and in turn facilitates and trigger char formation of PVA chains.

**Table 3:** The flammability properties of pristine and developed polymer nanocomposites.

Sample Code	Burning distance (mm)	Time (S)	Rate of Burning (mm/min)	Reduction (%)
PVA-MLNP-0 PVA-MLNP-10	75 75	36 79	125 57	0 54
PVA-MLNP-20	60	117	31	75
PVA-MLNP-40	25	40	37.5	70
PVA-MLNP-50	0	0	0	100

**Fig. 7:**
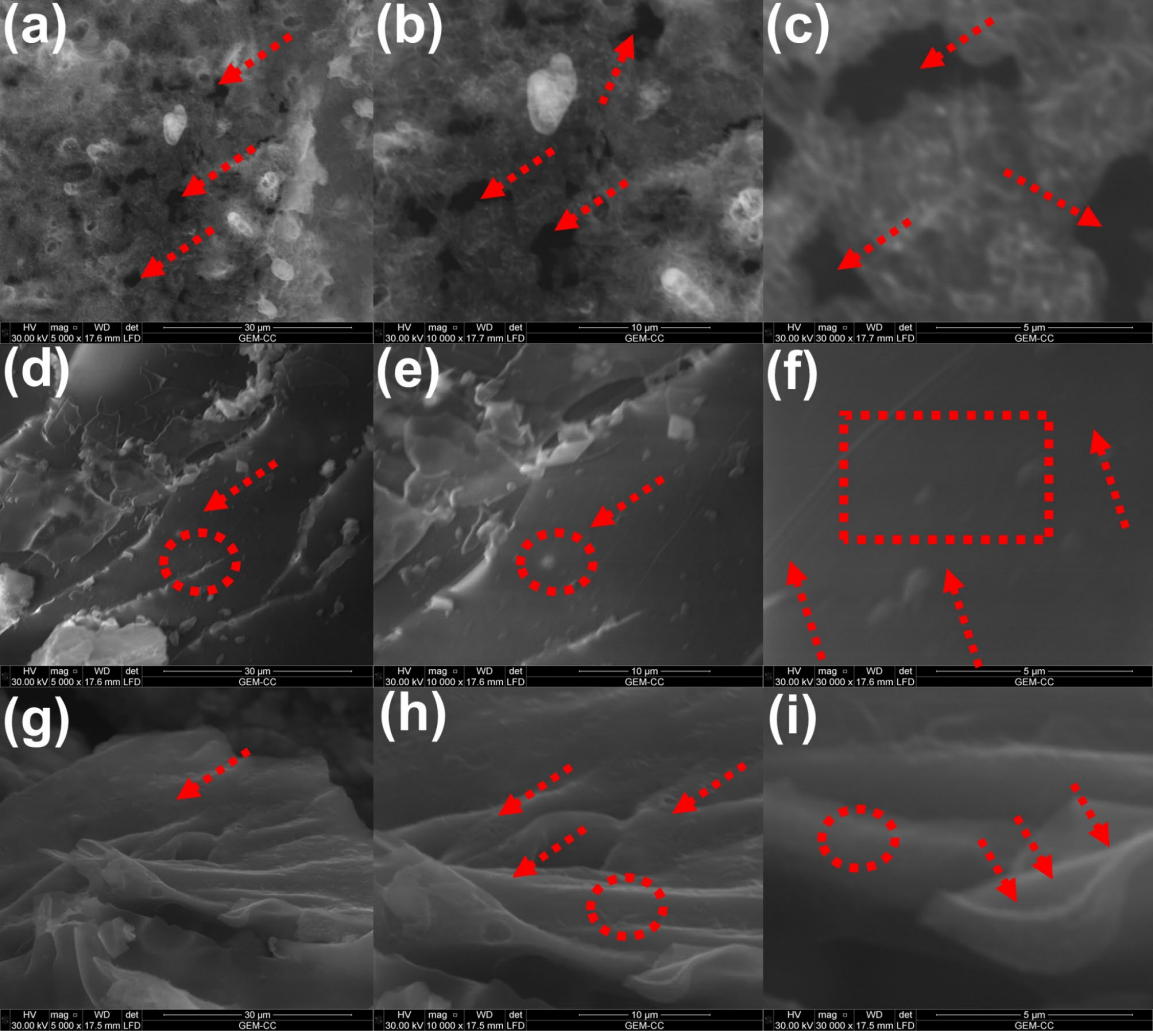
SEM image of char layer obtained after burning of PVA (**a**), SEM image of char layer obtained after burning PVA at high magnification (**b**-**c**), (Red arrows reflecting the broken and pores), SEM image of char layer after burning of PVA-MLNP-40, (**d**) SEM image of char layer after burning of PVA-MLNP-40 at high magnification (**e**-**f**) (Red arrows and circles displaying dense char layer obtained), SEM image of char layer after burning of PVA-MLNP-50 (**g**) and SEM image of char layer after burning of PVA-MLNP-50 at high magnification Red arrows and circles displaying dense char layer obtained).

The flame retardancy mechanism was further elucidated via investigation the morphology and elemental composition of char layer obtained after UL94 test of PVA-MLNP-0, PVA-MLNP-40 and PVA-MLNP-50. Figure 7a represents the SEM image of char barrier formed after burning of PVA (PVA-MLNP-0) alone which displayed broken char layer rich with porous structure which facilitates the easier escape of combustible gases and in turn feed the flaming zone and enrich combustion process of sample^33,44–50^. The porous structure formed on char layer was further elucidated at high magnification SEM images (Fig. 7b-c). Additionally, the elemental composition of this char layer was found to be only carbon and oxygen (Table 4 and Fig. S3). In the contrary, the protective char layer obtained after burning of PVA-MLNP-40 was found to be denser and compact of graphitic structure without any noticeable pores or broken layer was attained as shown in Fig. 7d-e. This supress the escape of combustibles gases and successfully isolated flaming zone from pyrolysis one and then retards mass and heat transfer (Fig. 7f)^33,44–50^. Interestingly, this protective char layer was enriched with precious elemental composition of various elements (Table 4 and Fig. S3) in addition to carbon and oxygen which synergistically enhanced the flame retardancy action (enhanced charring tendency) in condensed phase action. Furthermore, these metals played a significant role for strengthen the mechanical properties of the obtained char layer. This flame retardancy action was further corroborated once mass loading of MLNPs increase. Thus, the SEM images of char layer of PVA-MLNP-50 visualized superior dense and compact structure of protective char layer which is strong enough to completely inhibits the mass and heat transfer and in turn self-extinguish the sample achieving higher fire safety (Fig. 7g-i). This superior flame retardancy action was ascribed to the higher mass loading of MLNPs which in turns affords higher percentage of precious elements (Si, P, Cl, K, Ca and Fe) compared to PVA-MLNP-40 and then provides superior charring ability and superior inducing for PVA chains for char formation (Table 4 and Fig. S3).

**Table 4:** Elemental composition of char developed from PVA-MLNP-0 and their nanocomposites.

Sample Code	PVA-MLNP-0	PVA-MLNP-40	PVA-MLNP-50
C (At. %)	42.17	61.70	48.21
O (At. %)	57.83	19.61	30.24
N (At. %)	0	13.06	13.71
Na (At. %)	0	0.72	0.82
Mg (At. %)	0	0.46	0.29
Si (At. %)	0	0.33	0.53
P (At. %)	0	0.26	0.32
Cl (At. %)	0	0.28	0.57
K (At. %)	0	2.65	3.15
Ca (At. %)	0	0.93	1.20
Fe (At. %)	0	0	0.12

**Fig. 8:**
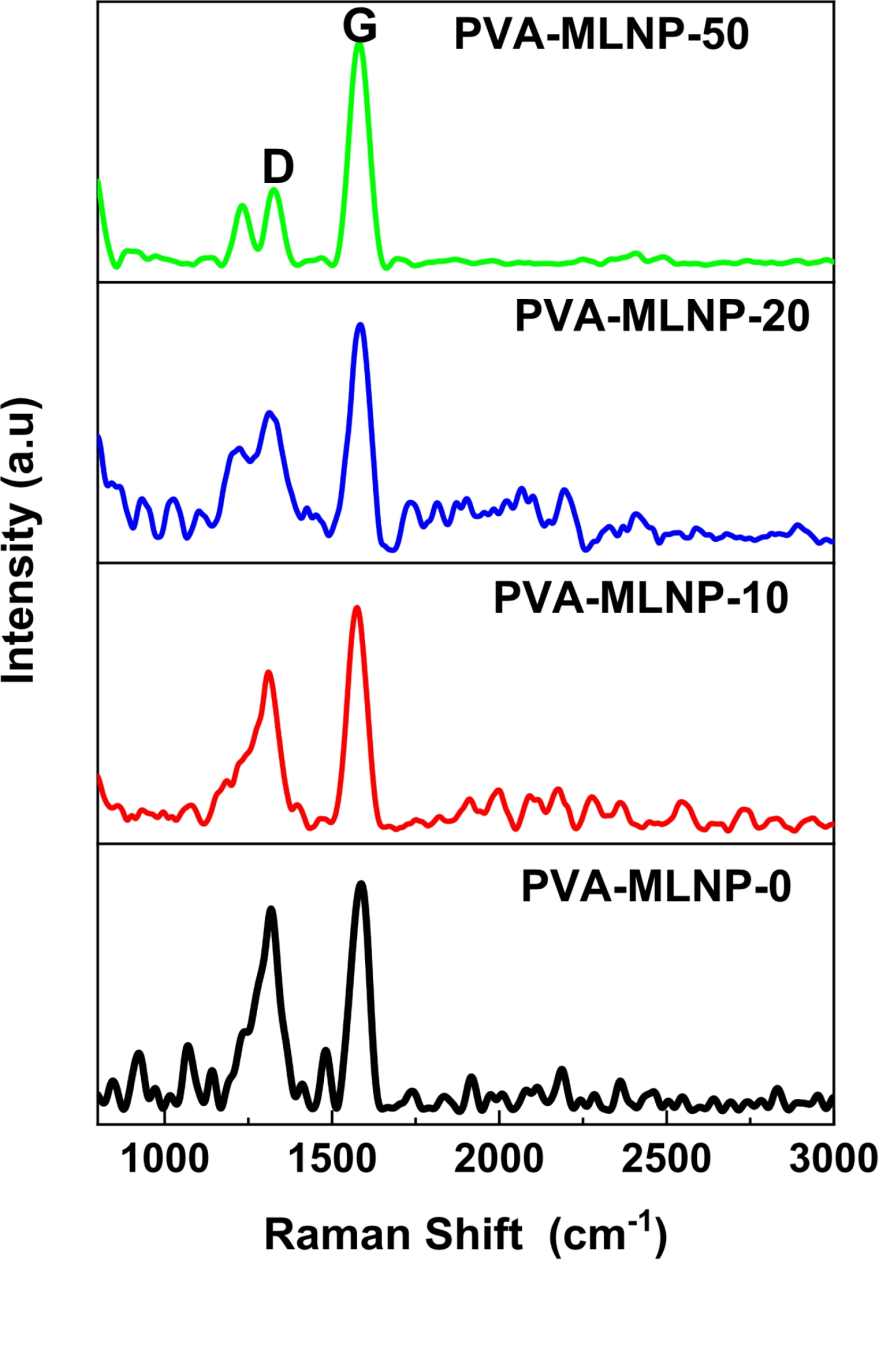
Raman spectra of PVA-MLNP-0, PVA-MLNP-10, PVA-MLNP-20 and PVA-MLNP-50.

Additionally, the efficiency of flame retardancy mechanism was further investigated by employing Raman spectroscopy to assess the quality of the graphitic structure of the protective char layer. Thus, Fig. 8 displayed the Raman spectra of char of PVA-MLNP-0 (blank PVA), PVA-MLNP-10, PVA-MLNP-20 and PVA-MLNP-50. For all spectra two strong bands were observed corresponding to the D and G bands, respectively (Fig. 8). These bands are suggestive of carbon-based materials and appeared nearly at 1330 and 1586 cm^− 1^, respectively. While the D band corresponds to the disorder of carbon structures, however, the G band belongs to sp^2^-bonded carbon atoms^51–53^. It was reported that, the intensity ratio of the D band *(ID)* over the G band *(IG)* determines the quality of the graphitic carbon structure of the protective char layer formed. Consequently, a larger *ID/IG* ratio indicates greater carbon disorder and structural instability, and vice versa, which is suggestive of superior flame-retardant properties^54,55^. Therefore, the graphitic char obtained from burning of PVA-MLNP-0 (pristine PVA) was discovered to have an *ID/IG* ratio of 0.87, but the graphitic char obtained from burning of PVA-MLNP-10 and PVA-MLNP-20 was determined to have an *ID/IG* ratio of 0.71 and 0.6, respectively (Fig. 8). Interestingly, the graphitic char obtained from burning of PVA-MLNP-50 was found to has superior quality of protective char layer recording *ID/IG* ratio of 0.32. This indicates that developed spherical MLNPs have a beneficial effect on producing higher graphitized char layer from PVA chains and in turn forming an excellent protective char layer. These results are consistent with the high flame retardancy characteristic that has been found, as shown in Table 3 (Figs. 7 and 8).

### UV protection, antibacterial and mechanical properties of developed nanocomposites

**Fig. 9:**
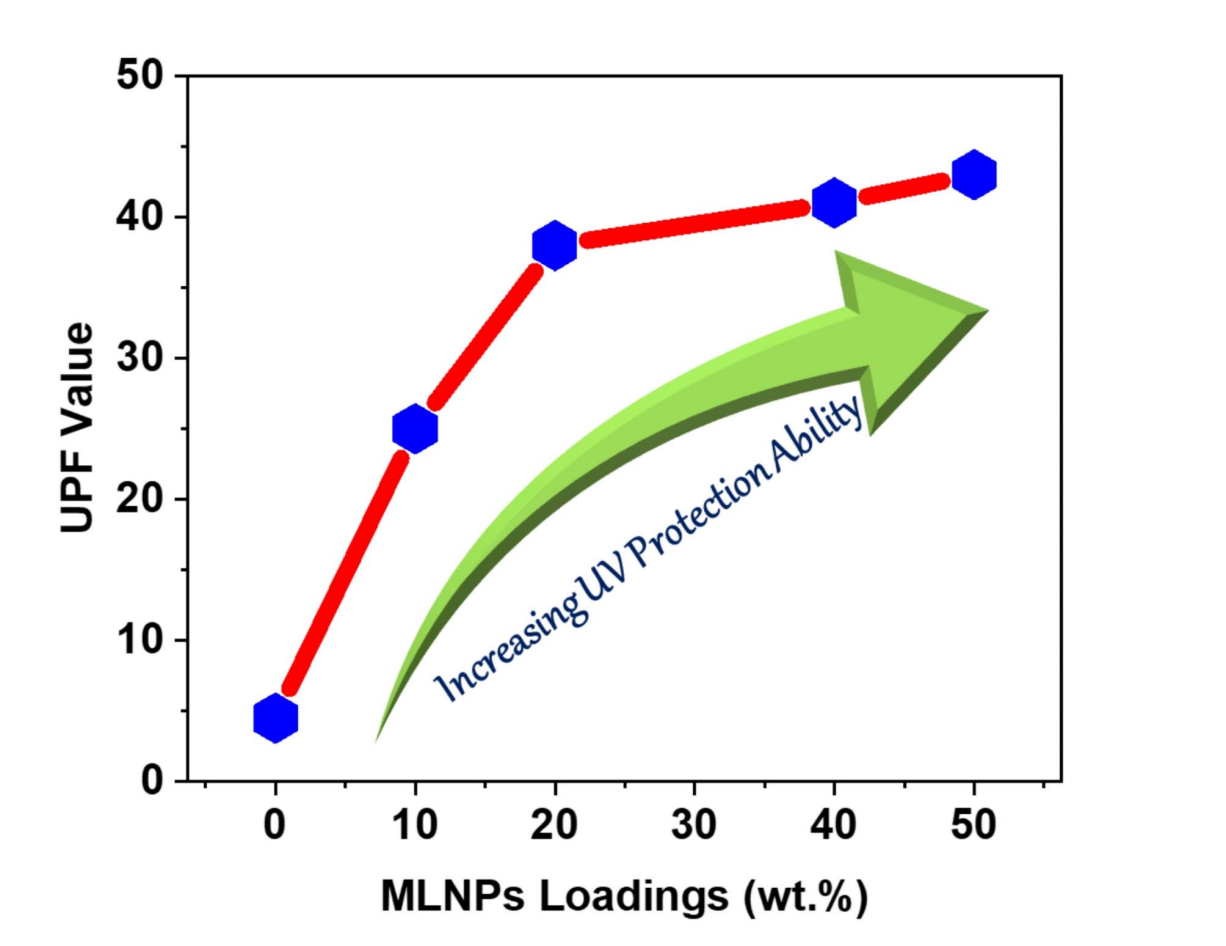
Correlation diagram displaying the UV protection increase against mass loadings of dispersed MLNPs .

Furthermore, because of its organic structure, the free-standing PVA thin film is highly susceptible to partial deterioration from exposure to intense harmful high energy UV rays. Additionally, the broad use of PVA in food packaging systems and medical applications was limited by the easier growth of microorganisms on its surface. Thus, to overcome these obstacles and increase the range of industrial applications for PVA, antibacterial and UV-protecting compounds should be incorporated in addition to flame retardant components for integrating high fire safety, UV protection and antibacterial properties for PVA films. Thus, the UV protection ability of the developed PVA nanocomposites were evaluated based on measuring UV protection factor (UPF) of blank PVA and their engineered nanocomposites, and the results were tabulated in Table 5 and shown in Fig. 9. The UPF value of blank PVA ; free MLNPs (PVA-MLNP-0) was found to be 4.5 displaying the high susceptibility of PVA toward negative impact of UV rays (Table 5). However, once incorporation of multifunctional MLNPs by 10 wt% (PVA-MLNP-10) a significant increase in UPF value was attained recording 25 and reflecting high UV protection ability as depicted in Table 5; Fig. 9. This indicates the strong capacity of MLNPs for absorbing the UV rays due to their chemical composition of various compounds rich with functional groups and Bi bonds^34,38,39^. This UV protection ability trend was obvious once the mass loadings of MLNPs were increased recording 38, 41 and 43 UPF values for PVA-MLNP-20, PVA-MLNP-40 and PVA-MLNP-50, respectively and reflecting superior UV protection ability in PVA-MLNP-50 as shown in Table 5; Fig. 9. Hence, the intrinsic ability of spherical MLNPs for UV absorption in conjunction with their uniform dispersion in PVA matrix afford promising UV rays protection properties for the developed PVA nanocomposite film and in turn expand their industrial applications.

**Table 5:** UPF values of PVA and their polymer nanocomposites films.

Sample Code	UPF
PVA-MLNP-0 PVA-MLNP-10	4.5 25
PVA-MLNP-20	38
PVA-MLNP-40	41
PVA-MLNP-50	43

**Fig. 10:**
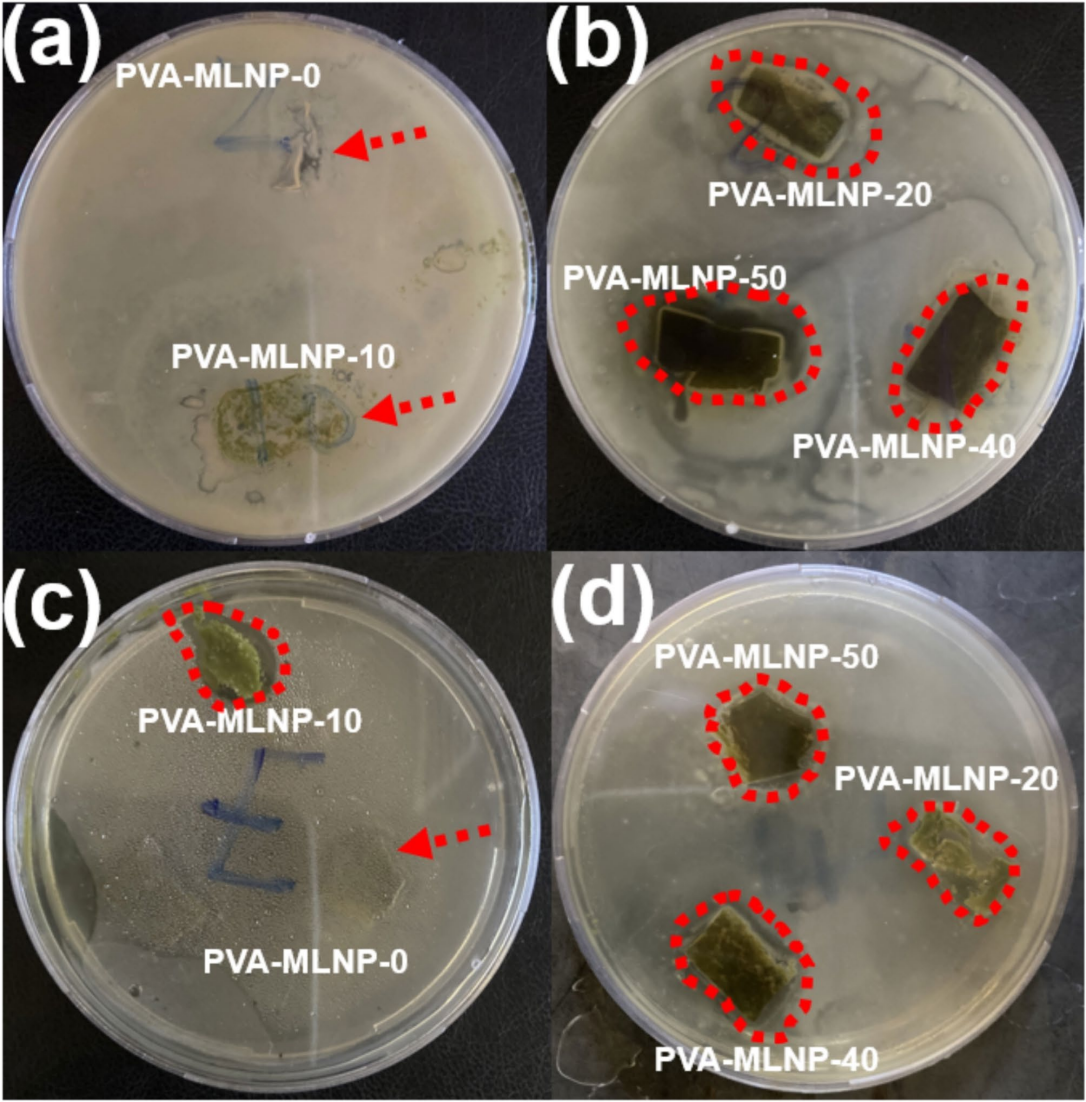
Digital photos displaying the clear antibacterial inhibition zone PVA and their developed nanocomposites against (**a**-**b**) *Staphylococcus aureus* and (**c**-**d**) *Escherichia coli*.

On the other hand, the inhibition of the bacterial growth on the surface of PVA and their developed nanocomposites were evaluated according to AATCC standard test method 147–2004^36^ against *Staphylococcus aureus* and *Escherichia coli* bacteria and the clear inhibition zone diameters and digital photos of antibacterial properties were shown in Table 6; Fig. 10. The inhibition of bacterial growth of pristine PVA (Free MLNPs) was found to be negative against the both bacteria types as indicated in Table 6; Fig. 10. On contrast, upon inclusion of 10 wt% of MLNPs to PVA for PVA-MLNP-10 nanocomposite, the antibacterial activity was significantly enhanced against gram -ve bacteria (*Escherichia coli)* recording clear inhibition zone of 9 mm, however no antibacterial action was noticed against gram + ve bacteria *(Staphylococcus aureus)* Table 6; Fig. 10. This obvious antibacterial activity for spherical MLNPs was ascribed to the rich antioxidants and phenolic compounds of their compositions which have antibacterial and anti-inflammatory properties^30–34^. Interestingly, once the mass loadings of MLNPs increased to 20 wt% (PVA-MLNP-20) obvious antibacterial activity was detected for *Staphylococcus aureus* achieving clear inhibition zone of 6 mm (Fig. 10). However, inferior clear inhibition zone (2.5 mm) was recorded for *Escherichia coli* as seen in Table 6; Fig. 10. For PVA-MLNP-40 nanocomposite, promising inhibition of bacterial growth was attained for both bacteria types achieving clear inhibition zone of 6 and 7 mm for *Escherichia coli and Staphylococcus aureus*, respectively (Table 6; Fig. 10). On the other hand, PVA-MLNP-50 recording superior inhibition of bacterial growth against *Staphylococcus aureus* (7.6 mm of clear inhibition zone), rather than *Escherichia coli* which achieved clear inhibition zone of 3 mm (Table 6). This difference in bacterial inhibition capacity of MLNPs at lower and higher mass loadings toward the two bacteria types is might be attributed to the difference of both bacteria types sensitivity toward MLNPs and in turn affect on their interaction and bacterial inhibition action. Therefore, based on this findings, PVA-MLNP-10 was found to be promising for inhibition of *Escherichia coli* bacteria growth achieving 9 mm clear inhibition zone, in the meanwhile PVA-MLNP-50 was found to be promising antibacterial *against Staphylococcus aureus* recording clear inhibition zone of 7.6 mm (Table 6; Fig. 10). The antibacterial properties were stemmed from MLNPs which containing abundant of anti-inflammatory and antioxidant (vitamin C, vitamin E, β-carotene, α-tocopherol, glutathione, and phenols)^30–33,56^. Which could afford metal ions chelation, thus, attributed to the release of cations from the bacterial cell envelope results in their sequestration, leading to changes in the cell wall structure. This alteration disrupts the selective permeability of the cytoplasmic membrane and ultimately causing metabolic failure^30–33^. Therefore, the scalable, innovative engineered bio-nanoparticles derived from ML provides higher safety against fire hazard, harmful UV rays and bacterial growth toward polymer nanocomposite films. Thus, this study, presents cost-effective route for production of sustainable industrial quantity of new generation of flame retardants, antibacterial and UV protecting agents for polymeric materials and promising for various electronics, engineering and food packaging applications.

**Table 6:** Bacterial clear inhibition zone diameters of PVA and their developed nanocomposites.

Sample Code	Clear inhibition zone (mm) Escherichia coli	Clear inhibition zone (mm) Staphylococcus aureus
PVA-MLNP-0	Zero	zero
PVA-MLNP-10	9 ± 1	Zero
PVA-MLNP-20	2.5 ± 0.8	6 ± 0.6
PVA-MLNP-40	6 ± 0.6	7 ± 0.7
PVA-MLNP-50	3 ± 0.5	7.6 ± 0.8

Interestingly, the mechanical properties of the PVA-MLNP-0 and PVA-MLNP-10 were carried out based on ASTM D 882 standard and tensile strength (TS), elongation at break (EB%) and elastic modulus (E-modulus) were recorded. Thus, the TS for PVA-MLNP-0 was found to be 24.35 MPa, however, the elongation at break was found to be 240%. This is in addition to E-modulus value of 11 MPa which displayed the ability of film for deformation resistant at elastic range. On the other hand, upon inclusion of spherical MLNPs in PVA-MLNP-10 nanocomposite film, the TS and EB% were found to be 22.61 MPa and 114.1%, respectively. However, the E-Modulus value was found to be 33.03 MPa. Although, the TS of PVA-MLNP-10 recorded less value than PVA-MLNP-0 by ~ 7%, however, superior E-modulus was attained for PVA-MLNP-10 (33.03 MPa) indicating superior resistant for developed nanocomposite film for deformation at elastic range. Therefore, this affords sustainable flame retardant, UV safe and antibacterial nanocomposite film for wide range of applications.

### Economic feasibility

From an industrial perspective, the MLNPs affordability and sustainability are essential components for its use in large-scale industrial applications. It is therefore very cost-effective to use ML as a renewable and green precursor for the production of MLNPs. Accordingly, considering the local cost of dried ML in Egypt, the solid-state synthesis process, and the annual availability of dried ML which exceed than 50,000 tone/year in Egypt. Furthermore, according to our method of synthesis and economic point of view of mass loading of MLNPs which achieved outstanding flame hazard safety, antibacterial, and UV protection properties (PVA-MLNP-40), it was found that producing of 1 Kg of MLNPs would cost approximately 1.4 US dollars.

## Conclusion

For the first time, an environmentally friendly one-pot solid-state ball-milling technique was used to engineer dry molokhia leaves into spherical nanoparticles with an average size of 8.5 nm. The sustainable nanoparticles were uniformly dispersed in PVA matrix yielding green well-dispersed polymer nanocomposites films. The mass loadings of developed nanoparticles were altered and optimized. Uniform dispersion of nanoparticles inside PVA matrix was attained due to the good compatibility between PVA chains and spherical nanoparticles leading to wrapping with PVA chains. The thermal stability, flame retardancy, antibacterial and UV protection of the as developed polymer nanocomposites were significantly improved. Higher fire safety for PVA nanocomposite achieving zero rate of burning compared to 125 mm/min for pristine PVA, this attributed to the unique chemical composition of nanoparticles which affords promising charring capacity which induces PVA chains for formation of well-strengthened protective char layer. The rich anti-inflammatory and antioxidant compounds in invented nanoparticles achieved outstanding inhibition to bacterial growth against well-known *Escherichia coli and Staphylococcus aureus* over the surface of developed polymer nanocomposites films. Furthermore, in comparison to nanoparticle-free polymer, the nanoparticles integrated exceptional UV protection feature of polymer nanocomposite films with a UV protection factor superiority of over 900%. Furthermore, an evaluation of the economic viability of manufacturing sustainable multifunctional nanoparticles at an industrial scale was carried out and 1 Kg could cost only 1.4 $ USA.

## Electronic supplementary material

Below is the link to the electronic supplementary material.

Supplementary Material 1

## Data Availability

The datasets used and/or analysed during the current study available from the corresponding author on reasonable request.
