# Supplementary material for: Innovative engineering of scalable, renewable and spherical organic nanoparticles for high fire safety, UV protection and antibacterial properties of polyvinyl alcohol nanocomposites films
Source: Sci Rep. 2024 Nov 21;14:28841. doi: 10.1038/s41598-024-80360-y (PMC11582320; doi:10.1038/s41598-024-80360-y)
Supplement: Supplementary file 1 — Supplementary Material 1 [file 41598_2024_80360_MOESM1_ESM.docx]

**Innovative Engineering of Scalable, Renewable and Spherical Organic Nanoparticles for High Fire Safety, UV Protection and Antibacterial Properties of Polyvinyl Alcohol Nanocomposites Films**

Nour F. Attia,^1*^ Mohamed A. Nour,^1^ Sally E.A. Elashery^2*^
 *^1^Gas Analysis and Fire Safety Laboratory, Chemistry Division, National Institute for Standards, 136, Giza 12211, Egypt*

*^2^Chemistry Department, Faculty of Science, Cairo University, Gamaa Str., 12613, Giza, Egypt*

*** Corresponding author:**

Prof. Dr. Nour Fathi Attia

*Gas Analysis and Fire Safety Laboratory, Chemistry Division, National Institute for Standards, 136, Giza 12211, Egypt*
E-mail: [drnour2005@yahoo.com](mailto:drnour2005@yahoo.com)


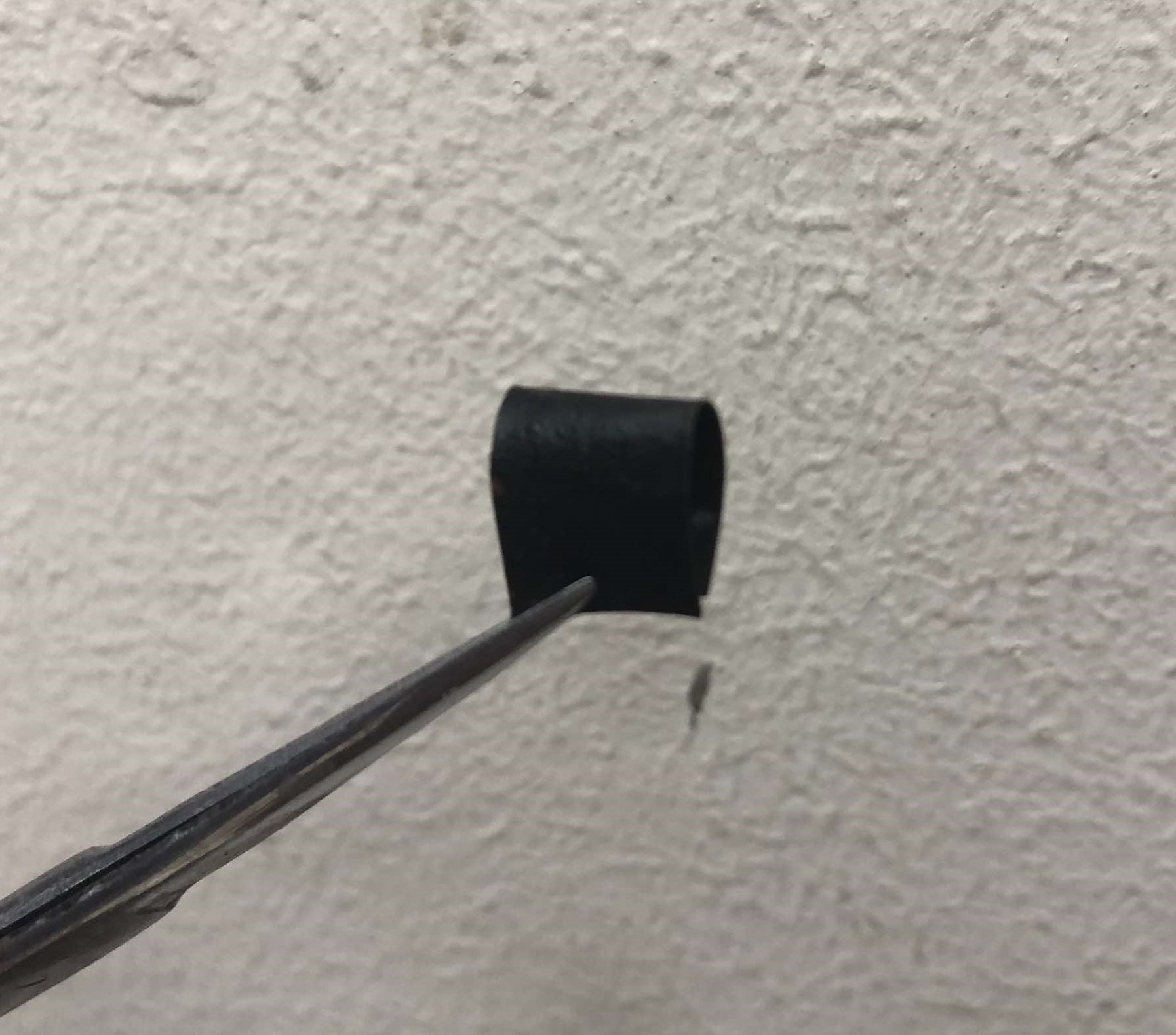


**Fig. S1.** Digital photo of PVA-MLNPs displaying the free-standing nanocomposite films.


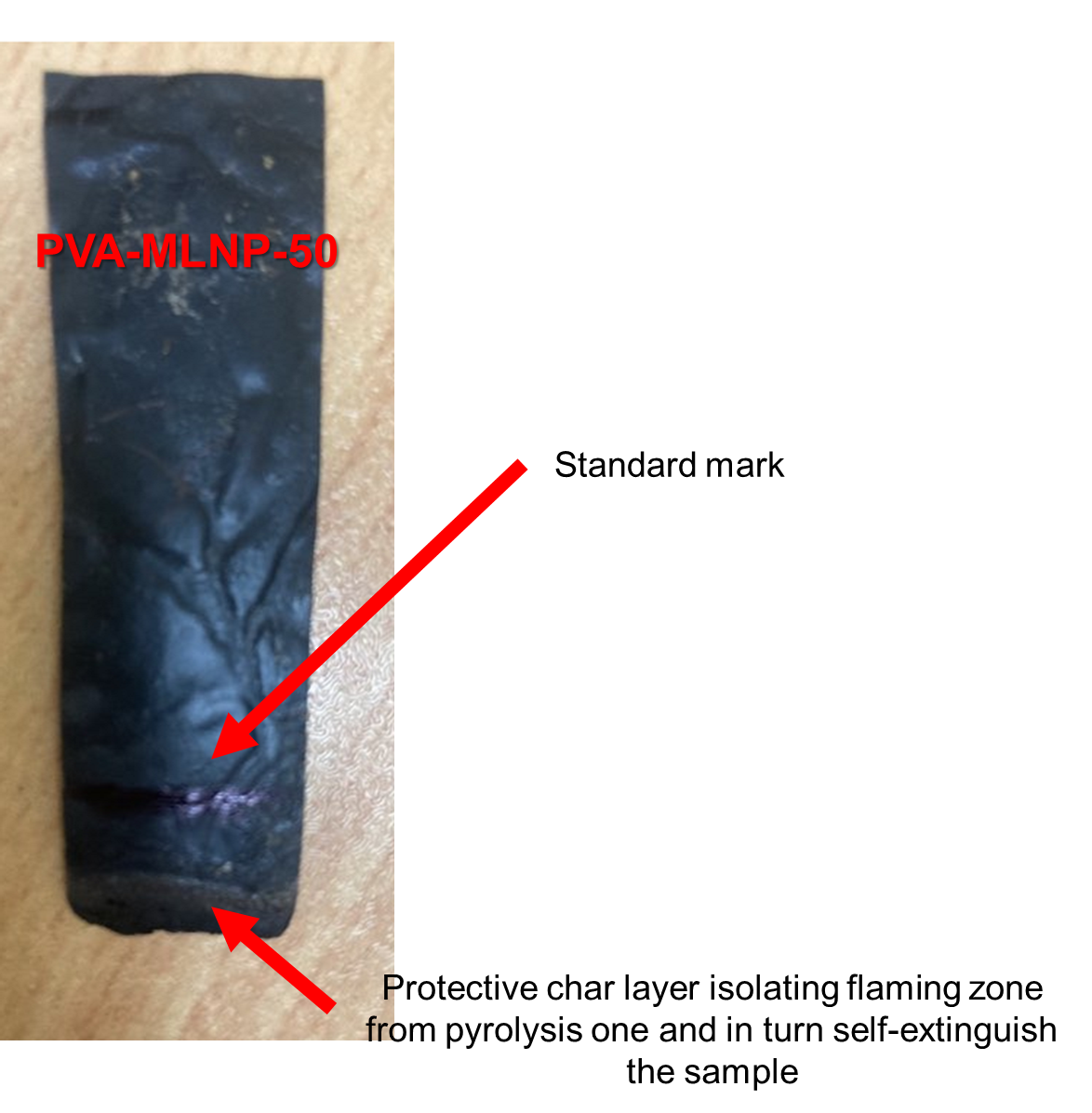


**Fig. S2.** Digital photo of PVA-MLNP-50 displaying the outstanding flame retardancy of free-standing nanocomposite film.


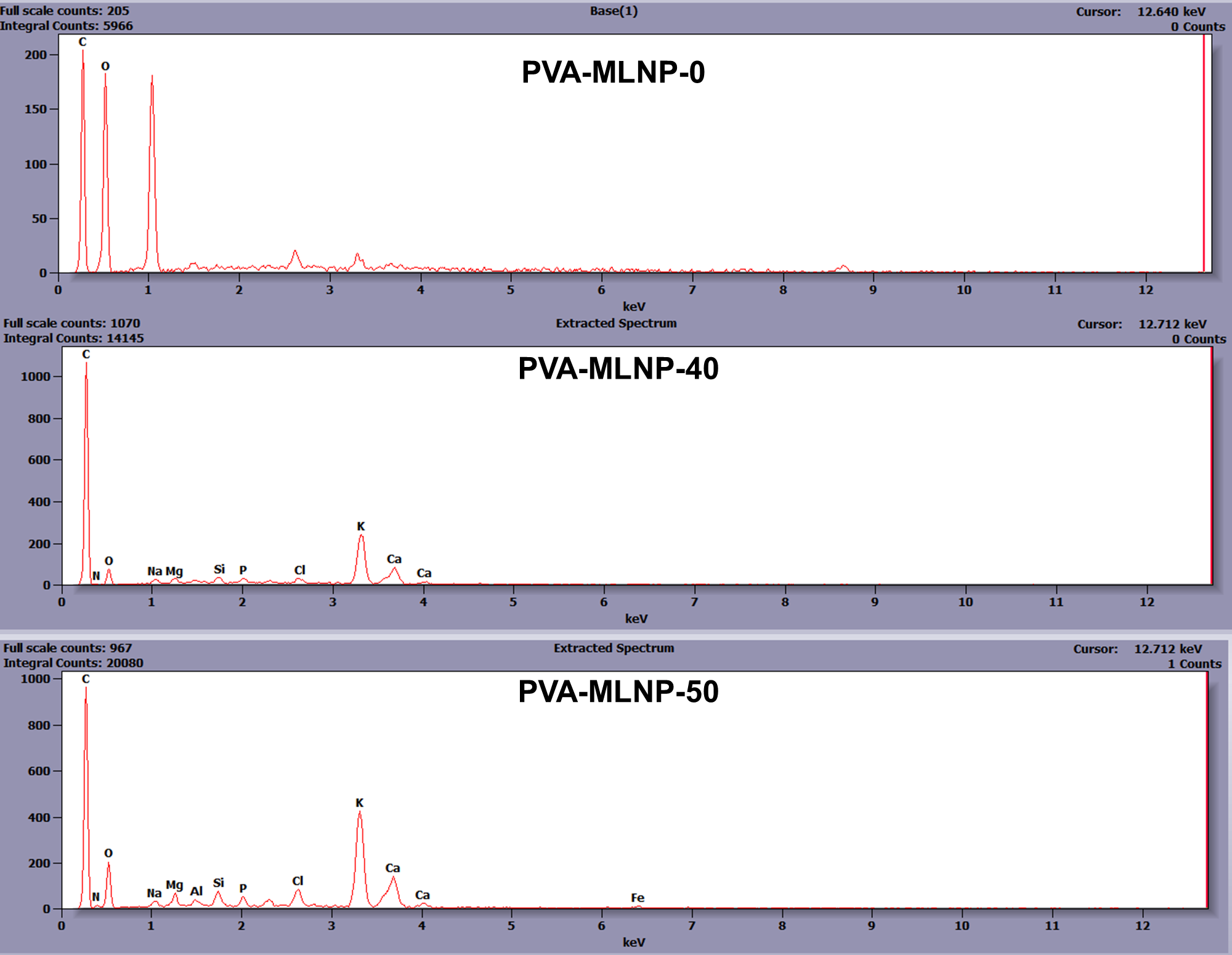


**Fig. S3.** EDS graphs image of char residue of PVA-MLNP-0 (blank PVA), PVA-MLNP-40 and PVA-MLNP-50 .
